# Prospects and challenges for the application of tissue engineering technologies in the treatment of bone infections

**DOI:** 10.1038/s41413-024-00332-w

**Published:** 2024-05-14

**Authors:** Leilei Qin, Shuhao Yang, Chen Zhao, Jianye Yang, Feilong Li, Zhenghao Xu, Yaji Yang, Haotian Zhou, Kainan Li, Chengdong Xiong, Wei Huang, Ning Hu, Xulin Hu

**Affiliations:** 1 Department of Orthopaedics, The First Affiliated Hospital of Chongqing Medical University, Chongqing, 400016 China; 2 Orthopedic Laboratory of Chongqing Medical University, Chongqing, 400016 China; 3 Clinical Medical College and Affiliated Hospital of Chengdu University, Chengdu University, Chengdu, Sichuan 610081 China; 4 University of Chinese Academy of Sciences, Bei Jing, 101408 China; 5 Department of Biotherapy, Cancer Center and State Key Laboratory of Biotherapy, West China Hospital, Sichuan University, Chengdu, Sichuan 610041 China

**Keywords:** Bone, Bone quality and biomechanics

## Abstract

Osteomyelitis is a devastating disease caused by microbial infection in deep bone tissue. Its high recurrence rate and impaired restoration of bone deficiencies are major challenges in treatment. Microbes have evolved numerous mechanisms to effectively evade host intrinsic and adaptive immune attacks to persistently localize in the host, such as drug-resistant bacteria, biofilms, persister cells, intracellular bacteria, and small colony variants (SCVs). Moreover, microbial-mediated dysregulation of the bone immune microenvironment impedes the bone regeneration process, leading to impaired bone defect repair. Despite advances in surgical strategies and drug applications for the treatment of bone infections within the last decade, challenges remain in clinical management. The development and application of tissue engineering materials have provided new strategies for the treatment of bone infections, but a comprehensive review of their research progress is lacking. This review discusses the critical pathogenic mechanisms of microbes in the skeletal system and their immunomodulatory effects on bone regeneration, and highlights the prospects and challenges for the application of tissue engineering technologies in the treatment of bone infections. It will inform the development and translation of antimicrobial and bone repair tissue engineering materials for the management of bone infections.

## Introduction

Bone infections are a group of diseases of the musculoskeletal system caused by microbial seeding via blood-borne or exogenous pathways.^1^ It mainly includes imgraft-associated osteomyelitis (periprosthesis joint infection, PJI), fracture-associated infection, acute blood-borne osteomyelitis and infectious bone defect.^2^ As the population grows and ages, the increasing prevalence of osteomyelitis will be a growing health problem. Since the 1970s, infection rates for open fractures have continued to increase to 5%–33%, while infection rates for joint replacements have increased to 1%–4%.^3,4^ Despite the use of the best medical care, osteomyelitis remains difficult to cure, with treatment failure rates ranging from 10% to 40%.^5,6^ Common pathogens that cause osteomyelitis include *Staphylococcus aureus* (*S. aureus*), hemolytic streptococcus, pneumococcus, Escherichia coli (E. coli), and Pseudomonas aeruginosa.^7^ Among them, *S*. *aureus* accounts for more than 70% of the pathogenic bacteria spectrum of osteomyelitis due to its strong invasion, colonization and osteocyte proliferation ability.^8^ Although surgical debridement combined with topical antibiotics and slow-release systems has significantly improved the cure rate of bone infections, the recurrence rate of chronic osteomyelitis is still as high as 20%–30%.^9^ Therefore, it is of great clinical value to elucidate the unique pathogenic mechanism of microorganisms in bone system and the disturbance of the biological process of bone regeneration mediated by microbial in the treatment of bone infection.

Actually, bone infection is usually accompanied by bone loss, so the treatment of bone infection needs to achieve rapid bone formation in the bone defect area in addition to effectively removing the pathogenic bacteria.^10^ Usually, prompt debridement combined with sensitive antibiotics can cure acute osteomyelitis. However, when it comes to chronic infection with bone defects, traditional treatment strategies are less effective.^11,12^ It is only when the infection is controlled that the repair of the bone defect begins.^13^ Changes in bone immune microenvironment induced by infection and local microenvironment induced by bone fillings are not necessarily conducive to bone repair biogenesis.^14^ The process of bone repair is not a simple process of bone formation and bone resorption, but a close interaction of multiple systems, including the skeletal system and the immune system, which is regulated by cytokines, stress stimulation and inflammatory response.^15^ Although bone transplantation, bone transport technology, bone induced membrane technology and antibiotic composite slow-release carrier implantation are widely used in the treatment of clinical bone infection, long treatment cycle, insufficient donor bone mass and complications in the donor area are urgent problems to be solved.^16,17^ Therefore, the development of bone-promoting bone fillings with antibacterial properties and adaptation to changes in the immune microenvironment of bone defects after infection will be an important therapeutic strategy with clinical significance. With the development of advanced material technology, tissue engineering technology is expected to become a solution to the coupling problem of infection control and bone regeneration and repair in infected bone defects. Tissue engineering involves the combination and synergy of cells, bioactive factors and scaffold biomaterials to further promote bone healing and regeneration based on improving the microenvironment at the site of locally infected bone defects.^18^ After the composite biological scaffold effectively filled the bone defect site, the release of antibacterial components can rapidly inhibit the proliferation of pathogens and prevent early excessive inflammation. At the same time, the osteogenic induction component can improve the damaged osteoblast ability and control the bone metabolism disorder caused by infection to promote bone repair.^19–21^ Therefore, a comprehensive understanding of the local microenvironment of infected bone defects is the basis for the design of these scaffolds. However, previous studies only focused on the function of scaffolds, and there was no comprehensive and in-depth analysis and discussion on the mechanism of infection in the process of bone defect repair.

**Fig. 1:**
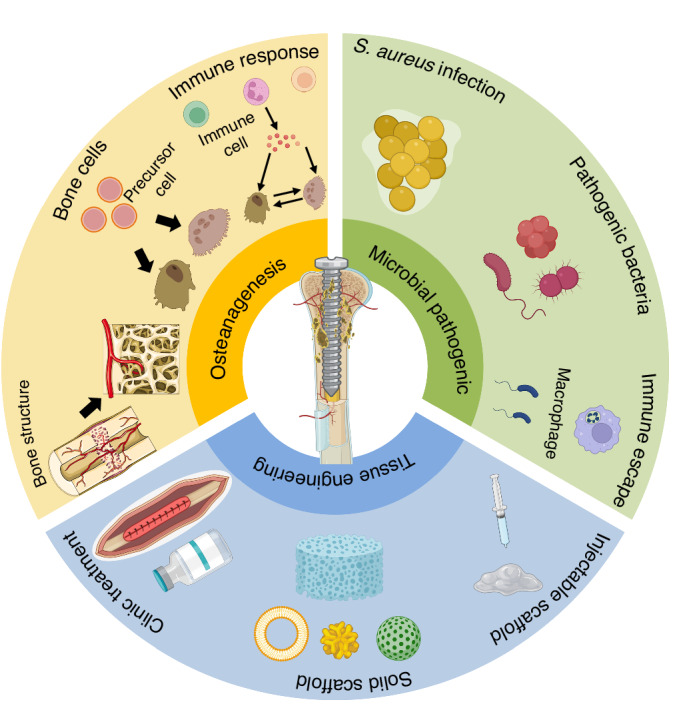
This review describes in detail the key pathogenic mechanisms of microorganisms in the skeletal system and their immunoregulatory role in bone regeneration, as well as the application of tissue engineering techniques in the treatment of bone infection

In addition, the immune system plays a crucial role in defending against bone infection and maintaining bone homeostasis, including mediating innate immune responses and adaptive immune responses to the skeletal microenvironment after pathogen invasion.^22,23^ Therefore, based on the biogenetic process of bone regeneration, various host immune escape strategies adopted by pathogens during osteomyelitis invasion were described in detail in this paper, and the mechanisms of immune activation, inhibition and regulation in the context of bone infection were introduced. At the same time, we discussed in detail the application of bone tissue engineering materials in the regulation of immune microenvironment and bone regeneration in bone infection, providing a novel and more promising therapeutic perspective for the clinical treatment of infectious bone defects (Fig. 1).

## Microbial infections and bone immune imbalance

### Pathogenic mechanisms of bone infections caused by pathogens

The primary mechanisms of *S*. *aureus* in bone infections include intracellular infection, OLCN invasion, biofilm and abscess formation.^3,24^ Studies have shown that the persistence and recurrence of osteomyelitis are associated with the intracellular infection of bone cells by *S*. *aureus*.^25^ *S*. *aureus* is capable of intracellular proliferation within a variety of cell types, including macrophages, keratinocytes, epithelial cells, endothelial cells, osteoclasts, osteoblasts, and osteocytes.^26–30^ However, current research on the intracellular persistence of *S*. *aureus* in osteomyelitis in vivo is limited. The intracellular infection of macrophages by *S*. *aureus* is often referred to as the “Trojan horse” phenomenon, which not only promotes bacterial dissemination throughout the body but also leads to the enrichment of small colony variants (SCVs).^26,31^ These SCVs exhibit metabolic changes, such as reduced metabolism and increased resistance to antimicrobial drugs, which are among the crucial factors contributing to the persistence and recurrence of infections.^31,32^ Currently, the three main mechanisms of *S*. *aureus* intracellular infection are believed to include survival within acidified phagosomes, formation of dormant SCVs to resist host defenses, and persistence within host autophagosomes.^3,24^ However, the detailed processes and life cycle of this infection are not fully understood, making further research of significant value.

It is commonly believed that large bacteria such as *S*. *aureus*, with diameters up to 1 µm, cannot penetrate the osteocyte lacunar canalicular network (OLCN)—a network of gap channels in dense bone structures with diameters of 0.5 µm connecting osteocyte lacunae.^33^ However, recent studies have shown that *S*. *aureus* can alter its shape and reduce its volume by nearly half, thus occupying lumens ranging from 100 to 600 nm in diameter.^34^ *S*. *aureus* has been found in the OLCN in both experimental infection models in animals and clinical cases, demonstrating its sub-micrometer level invasion of bone.^35–39^ This capability of *S*. *aureus* to invade bone at the sub-micrometer level may enable it to survive for extended periods and evade immune cells, leading to the inability to achieve bactericidal concentrations within the OLCN when high doses of antibiotics are applied systemically or locally.^24^ Therefore, designing innovative therapeutic approaches targeting OLCN invasion is crucial for effectively combating it.

Biofilms are structured communities of bacteria that mimic membranes and are associated with the extracellular matrix (ECM).^40^ They form when bacterial colonies attach and secrete extracellular polymeric substances (EPS), including polysaccharides, nucleic acids, and proteins, during their growth.^41^ Biofilms are common in infections related to medical devices, such as prosthetic joint infections (PJI),^42^ and other synthetic implant-associated infections.^43^ These biofilms act as barriers, creating a stable environment for bacterial activity and protecting bacterial cells from extreme conditions such as high temperatures, nutrient scarcity, dehydration, and antimicrobials.^44^ The inherent resistance mechanisms of biofilms pose challenges in treating deep infections, offering further protection to pathogens against antimicrobial substances and host immune responses.^45^ The formation of bacterial biofilms is a complex, three-dimensional process involving adhesion, aggregation, maturation, and dispersal, regulated by systems such as Agr and LuxS/AI-2.^3,46^ Initially, *S*. *aureus* adheres to surfaces using factors like cell wall proteins, adhesins, eDNA, and microbial surface components recognizing adhesive matrix molecules (MSCRAMM).^47–49^ After attachment, bacteria produce EPS for protection and growth.^46^ During aggregation, biofilms thicken in response to environmental cues.^50^ Mature biofilms form dense, mushroom-shaped structures with channels allowing the transfer of nutrients and oxygen throughout the biofilm.^51^ Finally, dispersal involves the breakdown of EPS and physiological changes, allowing cells to revert to a planktonic state and colonize new areas, continuing the biofilm cycle.^52,53^

During the progression of osteomyelitis, *S*. *aureus* forms unique staphylococcal abscess communities (SACs).^54^ Specifically, *S*. *aureus* attacks immune cells, such as neutrophils, and collaboratively creates a fibrin pseudo-capsule using coagulase and von Willebrand Factor (VWF).^55^ This fibrin pseudo-capsule acts as a protective barrier and attracts immune cells, many of which undergo necrosis. The accumulation of necrotic cells inadvertently serves as a barrier, limiting the entry of new immune cells and forming a bacterial sanctuary. This barrier ensures the long-term survival of the bacteria, rendering standard treatments ineffective. Consequently, the bacteria persist, and natural recovery is rare without targeted antimicrobial intervention.^56^ Simultaneously, the development of new drugs to eliminate the fibrin pseudo-capsule represents an effective approach to treating *S*. *aureus*.

In addition to *S*. *aureus*, several less common pathogens can also cause osteomyelitis, such as coagulase-negative staphylococci, Streptococcus species, Enterococcus species, and others. These pathogens can also evade antibiotics and the host immune system through mechanisms such as biofilm formation or the development of small colony variants (SCVs). For instance, Staphylococcus epidermidis can secrete SesC, Embp, and Sbp to aid in biofilm formation and stability.^57–59^ The fimbrial structures of Streptococcus agalactiae, with subunit proteins PilB and PilA, play a crucial role in its biofilm formation.^60^ Enterococcus faecalis’s quorum-sensing(QS) regulatory system, related to fsrA, fsrC, and gelE genes, and the three quorum-sensing systems in Pseudomonas aeruginosa—LasI-LasR, RhlI-RhlR, and PQS-MvfR—as well as genes like RpoS and EPS in Escherichia coli, contribute to the formation of mature and stable biofilm structures.^61–64^ Salmonella species, Brucella species, and Neisseria gonorrhoeae can mediate the persistence and recurrence of infections through the formation of SCVs.^32^ Rare pathogens in specific environments or populations can also cause osteomyelitis. In populations with immunosuppression due to treatment or various reasons, there have been reports of osteomyelitis caused by fungal infections such as Listeria, Weissella, Candida albicans, Aspergillus, and Cryptococcus neoformans.^65,66^ In pediatric osteomyelitis, bacteria such as Haemophilus influenzae, Kingella kingae, and Salmonella species have been detected.^67^ Coccidioides, common in the southwestern United States and Mexico, and Blastomyces, prevalent in the central and southern United States, may have higher local incidence rates.^66^ Salmonella species are commonly found in osteomyelitis associated with sickle cell anemia.^68^

### Bone immune imbalance and its impact on bone regeneration

**Fig. 2:**
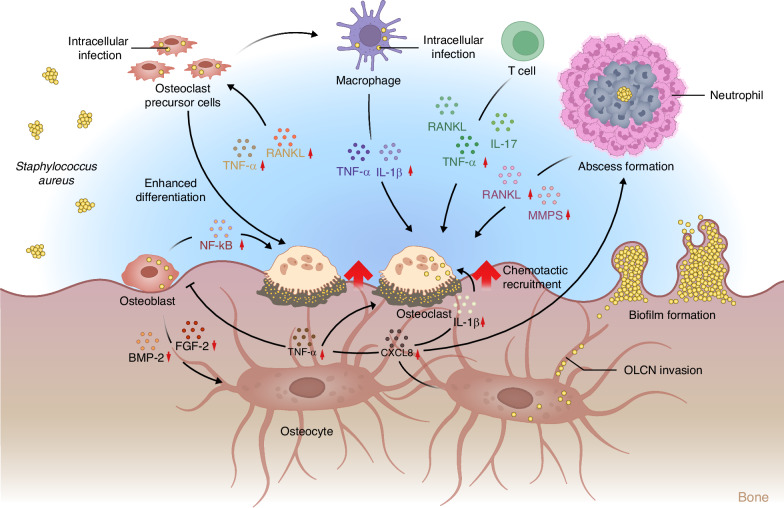
Effects of inflammation imbalance caused by microorganisms on bone immune microenvironment. In the context of chronic inflammation of the bone microenvironment due to persistent pathogen infection, osteoblasts upregulate the release of IL-1β and TNF-α through CXCL8. TNF-α inhibits the expression of osteogenic factors such as BMP-2 and FGF-2, while promoting the differentiation of osteoclast precursors into mature osteoclasts. IL-1β further enhances the maturation of osteoclasts. Additionally, CXCL8 recruits neutrophils, which are primary producers of MMPs responsible for the degradation of bone and cartilage. Under conditions of persistent infection, osteoblasts also stimulate the activation of NF-κB, promoting osteoclast formation and bone destruction. Furthermore, T cells influence the activity of osteoclasts and osteoblasts by secreting factors such as RANKL, TNF-α, and IL-17. Osteoclast precursors differentiate into osteoclasts and macrophages under the stimulation of RANKL and TNF-α. Macrophages stimulate the maturation and activity of osteoclasts through TNF-α and IL-1β

When pathogens invade bone tissue, the immune system is activated, releasing inflammatory factors and cytokines. These factors can enhance the activity of osteoclasts, leading to increased bone resorption, while simultaneously inhibiting the function of osteoblasts, thereby hindering the biological process of bone regeneration (Fig. 2).^69^ It is noteworthy that the mechanism of bone regeneration in an infectious environment involves molecular interactions between pathogens and immune cells as well as among bone cells themselves. Clarifying these interconnections holds significant clinical value. In the chronic inflammatory environment caused by infection, the impact of immune cells on bone regeneration cannot be overlooked. Macrophages, significant sources of cytokines, promote the production of tumor necrosis factor-alpha (TNF-α), IL-1β, and interferon-alpha (IFN-α) upon recognition of pathogen-associated molecular patterns by Toll-like receptors. These inflammatory factors activate vascular endothelial cells and lymphocytes, disrupting local tissues to increase the ingress of effector cells, and induce endothelial cells to form P-selectin, enhancing the recruitment of neutrophils.^70,71^ When *S*. *aureus* invades the body, the reactive oxygen species (ROS) produced by macrophages activate the NOD-like receptor pyrin domain-containing protein 3 (NLRP3), releasing active caspase-1, activating IL-1β and IL-18, and mediating pyroptosis.^72^ Under pathogenic stimulation, macrophages highly express pro-inflammatory factors to resist infection and activate the immune response. However, the prolongation of infection leads to sustained macrophage-mediated inflammation, inhibiting the formation and maturation of new blood vessels and reducing the healing speed of bone tissue, while continuously increasing osteoclast activity and inhibiting osteoblast differentiation.^73^ Neutrophils, highly active phagocytes, mediate the earliest immune response to pathogens. Upon infection, neutrophils are rapidly recruited from the bloodstream and migrate through endothelial cell interstices to the site of infection, driven by chemotactic factors produced by host cells and bacterial proteins. Pathogens phagocytosed by neutrophils are isolated in vesicles and eliminated by ROS and degranulation produced by neutrophils. Neutrophils can also exert antibacterial activity through the formation of extracellular traps (NETs).^74^ However, the high concentrations of oxidants produced by neutrophils often result in abnormal inflammatory side effects. The accumulation of neutrophils in the peri-inflammatory tissues of bones, along with high levels of reactive oxygen species, nitric oxide synthase, and NETs, promotes the disruption of the oxidative-antioxidative balance, leading to cytotoxicity, tissue damage, and bone loss. Moreover, neutrophils highly express RANKL under pathogen virulence factors such as LPS stimulation, thereby stimulating osteoclast formation. The inflammatory factors produced by immune cells are also linked to innate immune cells. Macrophages producing IFN-α and IL-12 can activate NK cells and induce CD4^+^ T cells to differentiate into TH1 cells. Although TH1 cells secrete IFN-γ and TNF-α to eliminate pathogens, their high expression of RANKL and TNF-α is a significant factor influencing osteoclast differentiation. IL-6 and TGF-β-induced TH17 cell maturation, and their production of IL-17, can also induce an increase in RANKL expression in osteoclast precursors and osteoblasts, thereby promoting bone resorption.^75^

During the bone regeneration process, acute inflammation leads to the production of chemokines, which also affect the differentiation of mesenchymal stem cells (MSCs) into osteoblasts.^76^ Studies have shown that under the persistent infection of *S*. *aureus*, the production of chemokines by bone cells significantly increases, including CCL5, CXCL1, CXCL8, CXCL9, CXCL10, and CXCL11. CXCL8 can upregulate the release of IL-1β and TNF-α.^76^ The increase in TNF-α can induce the differentiation of pre-osteoclasts into mature osteoclasts and cause the activation of NF-kB in osteoblasts and osteocytes, thereby inhibiting the expression of osteogenic factors such as BMP-2 and Fibroblast Growth Factor 2 (FGF-2). IL-1β can further promote osteoclastogenesis through c-Jun N-terminal kinase (JNK) and NF-κB pathways.^73^ Simultaneously, CXCL8 can chemotactically recruit neutrophils, which are major producers of matrix metalloproteinases (MMPs) involved in the degradation of bone and cartilage. The increase in MMPs, along with the activation of osteoclasts, ultimately leads to bone resorption and destruction. Osteoblasts in a persistent infection can also release inflammatory factors and chemokines. Studies have shown that after *S*. *aureus* infection, the release of osteoblast IL-6 and the activation of NF-κB receptor activator of nuclear factor kappa-Β ligand (RANKL) increase. Similar to IL-1β, IL-6 induces osteoclastogenesis and bone destruction. RANKL, a member of the TNF superfamily, is a primary effector molecule for the survival, proliferation, and differentiation of osteoclasts. It can bind to NF-κB and induce the differentiation of monocyte/macrophage lineage cells into osteoclasts, further leading to the maturation of osteoclast precursors.^76^ Trouillet-Assant et al. found that pathogen infection of osteoclast precursor cells could induce differentiation into macrophages, releasing more pro-inflammatory factors such as macrophage inflammatory protein 1α (MIP-1α), CCL2, and CCL5. Osteoclast precursors in this environment are more likely to differentiate into osteoclasts under the co-culture of RANKL and M-CSF.^77^

Therefore, although the inflammatory environment caused by infection is beneficial for clearing pathogens, its dysregulation and the resultant cytokine induction often have detrimental effects on bone regeneration.

## Tissue engineering treatment of bone infections

The repair and treatment of infected bone defects is a common challenge in multidisciplinary fields such as orthopedic clinics, biomaterials science, and tissue engineering.^78,79^ Presently, autologous, or allogeneic bone grafting is widely regarded as the optimal approach for addressing bone defects. Nonetheless, its clinical implementation is often hindered by constraints such as scarcity of donors, donor site discomfort, infection, and hemorrhaging.^80,81^ Furthermore, the administration of systemic antibiotics is associated with drawbacks such as inadequate local drug concentration for combating infections, susceptibility to drug resistance, and systemic adverse effects.^82,83^ Therefore, there is an urgent need for a new treatment method in current clinical medicine to break through the existing methods and achieve the purpose of first-stage treatment. With the rapid development of cellular technology, biomimetic materials, and microsurgery technology in recent years, bone tissue engineering (BTE) has also made great progress. Bone repair scaffolds are the core components of bone tissue engineering because all other components need to be loaded onto the scaffolds to function. The development of new bone repair materials based on bone tissue engineering with excellent mechanical properties, high osteogenic activity, and local anti-infection has become a hot spot of research attention.

### Clinical treatment strategies for bone infections

The aim of treating bone infections is to thoroughly eliminate the infection focus, ensure soft tissue coverage, facilitate the healing of bone ends, and preserve the length and functionality of the limb. After the complete removal of the infection focus, bone defects often occur, which are sites where infection is prone to recur. The histological and biomechanical reconstruction of these defects is a lengthy process and has become a focus of osteomyelitis treatment research.^84^ Currently, there are several bottlenecks in the treatment of bone infections,^3,25,27,85^ such as: (1) the formation of multidrug-resistant bacteria reducing the efficacy of antibiotics; (2) bacterial infections in bone tissue can form biofilms and adhere to dead bone and implant surfaces for a long time, serving as a “shield” for bacteria against immune responses and antibiotics; (3) infected phagocytes (such as macrophages) become “Trojan horses,” protecting bacteria from the lethal effects of antibiotics following immune responses, ultimately leading to persistent infections and recurrent relapses. The primary clinical treatments for infectious bone defects include surgery and antibiotic therapy.^86^ Additionally, 3D-printed bone implants, with their excellent bactericidal properties, strong mechanical support capabilities, and potent bone induction abilities, have emerged as a promising treatment option in the field of infectious bone defect repair.^87,88^

Adequate surgical debridement is a prerequisite for the treatment of bone infections, and its effectiveness often depends on anatomical location, soft tissue integrity, the presence of implants, the presence of deep abscesses or biofilms, the host’s immune status, and the infecting microorganisms.^89,90^ When local infection is controlled, the repair of local bone defects must be considered. Currently, the repair of infectious bone defects primarily includes bone grafting, the Ilizarov technique, and the Masquelet technique, each with its advantages and disadvantages were shown in Table 1.^86,89,91^ Moreover, selecting sensitive antibiotics is fundamental to treating bone infections, requiring high doses, sufficient treatment duration, and combination therapy. Traditionally, antibiotic treatment is believed to last 4 to 6 weeks, based on animal studies and the approximately 4 weeks required for revascularization after bone debridement.^10,92^ However, the unique invasion and colonization mechanisms of pathogens in the skeletal system increase the difficulty of treating bone infections with traditional antibiotics, especially intracellular infections and those involving the OLCN.^24^ Lehar and colleagues developed an antibody-antibiotic conjugate for treating intracellular infections.^93^ This conjugate can bind to the cell surface and be phagocytosed into lysosomes, where active antibiotics are released under the action of proteases to kill intracellular pathogens.^94^ Antibiotic treatment at the site of chronic osteomyelitis can, to some extent, compensate for the shortcomings of systemic application, but it remains controversial. Methods for local antibiotic use include direct placement of drugs at the infection site, closed irrigation therapy, interventional plus extracorporeal micro-pump, and local antibiotic release systems. Currently, local antibiotic slow-release systems are the most common method of local administration for treating chronic infectious bone defects. Polymethylmethacrylate (PMMA) drug delivery systems are the most widely used local antibiotic slow-release systems in clinical practice. However, as clinical use and research on PMMA have advanced, its limitations have gradually emerged: (1) The decline in drug concentration to levels insufficient for bactericidal action can facilitate the formation of resistant bacteria; (2) Secondary surgery to remove the material increases the risk of surgery and infection; (3) The drug release concentration of PMMA is difficult to precisely control, and release is not complete. Therefore, there is an urgent clinical need for a new material that not only possesses the supportive strength, biocompatibility, and biosafety of bone cement but also offers more efficient antibacterial action and promotes bone repair.^94,95^

**Table 1:** Advantages and disadvantages of surgical strategies

Surgical Strategies		Advantages	Disadvantages
Bone graft	Autogenous bone graft	(1) Good bone conduction, osteoinduction, adhesion and permeability. (2) Low infection rate. (3) Conducive to the growth of blood vessels.	(1) Large trauma and excessive bleeding. (2) Sources are limited. (3) Taking autologous bone increases the chance of cross-infection.
	Allogeneic bone graft	(1) The operation is simple and short. (2) No additional surgical trauma. (3) Sufficient sources.	There is a risk of infection, incomplete absorption, disease transmission and immune rejection may occur.
	Free vascularized fibulagraft	(1) Conducive to the reconstruction of blood circulation. (2) Sufficient length to provide bone mass. (3) Healing speed is fast. (4) The hard bone of the fibula can provide sufficient support.	(1) High technical requirements for microsurgery. (2) Poor survival of blood vessels leads to an increase in surgical failure rate.
Masquelet technique		(1) The operation time is short and there are few postoperative complications. (2) It can be used to treat large infected bone defects. (3) Antibiotics - bone cement continuously releases antibiotics to control infection. (4) Induce the formation of fibrous membrane and provide good blood supply. (5) A relatively independent and stable space is formed in the bone defect, which is conducive to second-stage bone reconstruction and bone healing.	(1) The surgery is invasive and requires a long period of time. (2) The induction membrane is easily damaged, leading to poor osteogenesis. (3) The ingrowth of soft tissue is uncontrollable, increasing the difficulty and trauma of surgery.
Ilizarov technique		(1) Small trauma, short operation time, few complications, and high success rate. (2) Controllable deformity correction, providing a foundation for later rehabilitation; (3) No secondary trauma. (4) The force is more uniform and there is no stress shielding.	(1) Complications related to bone and soft tissue may occur, such as skin cutting, malalignment, premature mineralization of new bone, poor mineralization of new bone, malunion or nonunion of fractures, joint stiffness, etc. (2) External fixator-related complications, such as external fixator loosening, nail track pain, nail track infection, etc. (3) The treatment and recovery cycle is long. (4) The operating procedures are complex and require regular adjustments. (5) It may lead to foot drop deformity.

### Scaffold for bone infection repair

#### Design of scaffold for bone infection repair

As mentioned in the previous section, the current stage of treatment for infectious bone defects mainly involves multi-stage surgical treatment, poor antimicrobial performance, and a lack of ideal bone grafts. In addition, the complex microenvironment of bone infection and the invasion of a large number of bacteria lead to the abundant attachment of bacteria on the surface of implants, resulting in a “competition for colonization” between bone cells and bacteria.^96^ Furthermore, changes in the microenvironment such as infection of extracellular matrix of bone cells, impaired bone formation, and increased osteoclasts also hinder the process of promoting bone regeneration by impeding the filling of grafts.^97^ Therefore, the urgent problem to be addressed is the selection of a filling scaffold that combines antimicrobial and bone-promoting functions to avoid secondary bone graft surgery and reduce the rate of infection recurrence.

**Fig. 3:**
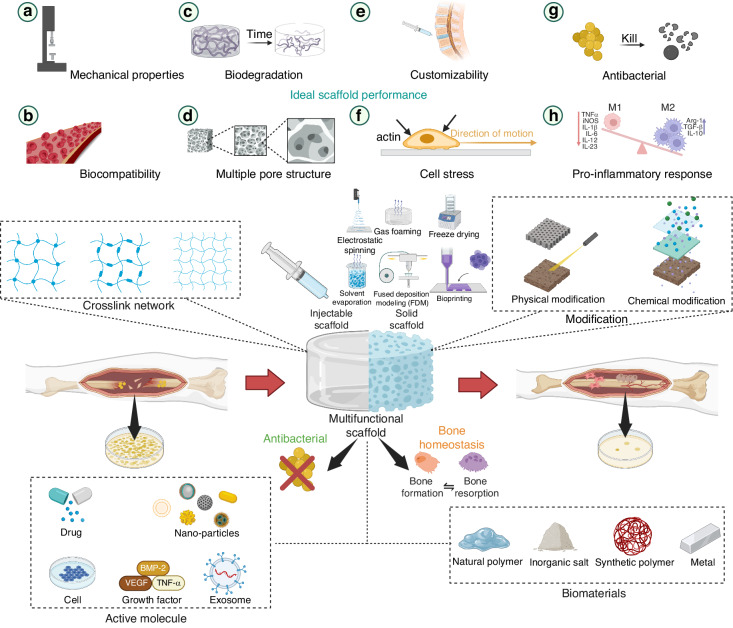
Construction of a multifunctional filling scaffold for bone infection. **a** Mechanical properties. **b** Biocompatibility. **c** Biodegradation. **d** Multiple pore structure. **e** Customizability. **f** Cell stress. **g** Antibacterial. **h** Pro-inflammmatory response

The current research on fillable scaffolds for infectious bone defects mainly involves tasks in material selection, scaffold construction, drug synthesis, and controlled release. Infectious bone defect fillable scaffolds can be divided into injectable scaffolds and solid scaffolds. Injectable hydrogel scaffolds are favored for their good formability, fillability in irregular bone defects, and good biocompatibility and drug loading capacity mimicking the extracellular matrix (ECM). On the other hand, solid fillable scaffolds can meet the mechanical requirements of the defect site and can be prepared in various ways, making it easier to mimic natural bone tissue from a structural performance perspective.^98,99^ Whether injectable or solid, both types of scaffolds need to consider the relationship between material composition and properties, biomimetic structure construction, antimicrobial properties, promoting bone function, and their interactions. An ideal repair scaffold for bone infections should possess the following characteristics (Fig. 3):

Under adequate mechanical performance, cortical bone and cancellous bone exhibit different mechanical properties. Compared to cancellous bone (compressive strength of 2 ~ 12 MPa, Young’s modulus of 7 ~ 30 GPa), cortical bone has greater compressive strength and stiffness (compressive strength of 130 ~ 230 MPa, Young’s modulus of 0.02 ~ 0.5 GPa). Therefore, when simulating bone tissue properties, the compression performance of the scaffold needs to be evaluated according to the filling site. In addition, bone defect patients have different requirements for bone tissue load-bearing capacity due to disease and age factors. Therefore, it is necessary to design bone substitutes with customizable and controllable mechanical strength.^100^ 2. The scaffold should not hinder cell proliferation and differentiation, and should be non-toxic to cells.^101^ 3. The transformation of artificial bone tissue substitutes to biological bone tissue needs to achieve a dynamic balance between degradation rate and new bone growth rate. 4. Customizable supports can maximize the fit to the defect site, such as achieving customized mechanical properties in the defect site and support structure during large-scale debridement of irregularly shaped infected bone defects. 5. For artificial bone scaffolds with degradable behavior, they should have a multi-layer pore structure to mimic the large and small pores of natural bone tissue. The interconnected large pore structure (>100 μm) can bear stress transfer functions, promote bone tissue ingrowth and extension. The number, size, and distribution of micro-pores usually have a significant impact on the metabolism and proliferation of bone tissue, with pore sizes of 150 ~ 800 μm facilitating nutrient transport and metabolic waste excretion. Pore sizes of 40 ~ 100 μm are beneficial for the growth of non-mineralized tissues.^102^ 6. For hydrogel-based scaffolds, by constructing scaffolds that simulate ECM components and structure, stimulation of cell mechanical responses can be achieved, thereby regulating downstream signaling pathways.^103^ 7. In addition to ensuring the conductivity, inductivity, and integration of scaffold bones, it is also necessary to consider regulating the immune microenvironment and promoting angiogenesis, as well as rapidly relieving and clearing specific pathological symptoms, while providing mechanical support for local lesions, ultimately achieving the goal of promoting bone regeneration.^104,105^

#### Selection of materials for bone infection repair

##### Inorganic materials for bone infection

In orthopedic surgeries, postoperative infection caused by filling metal prostheses is the most serious and common problem, often accompanied by local inflammation. Due to their bioinertness, these metal prostheses often fail to provide a favorable platform for bone unit integration and osteogenic differentiation.^106,107^ However, inorganic metal particles represented by gold, silver, zinc, and copper have been widely used in composite anti-infection scaffolds. Metal particles, due to their extensive physicochemical characteristics including size, charge, zeta potential, surface morphology, and structure, can have a significant impact on bacteria.^108^ In this process, metal particles can attach to the bacterial membrane through electrostatic interactions, van der Waals forces, receptor-ligand or hydrophobic interactions, penetrate the bacterial membrane, and primarily affect bacterial normal functions through the following pathways: (1) Metal particles can induce cell membrane damage, primarily due to the interaction between metal ions and bacterial membrane lipids, causing changes in membrane charge after binding with cations, leading to unstable rupture of lipid membrane, resulting in bacterial death due to imbalanced osmotic pressure and limited ability to transmit signals.^109^ (2) Destruction of bacterial proteins and DNA occurs within the bacteria by metal particles disrupting metabolic pathways, catalyzing the oxidation of specific amino acids, reducing protein stability, and marking protein degradation.^110^ (3) Reactive oxygen species (ROS) are generated, and active redox-active metals can typically convert hydrogen peroxide into highly toxic hydroxyl radicals through the Fenton reaction, leading to massive production of ROS to mediate bacterial death.^111^ At the same time, metal particles can greatly increase the selectivity of materials through the construction of composite scaffold systems by blending with the scaffold substrate, coating preparation, and material modification grafting.^112^

In inorganic materials, in addition to metal particles, bio-ceramics represented by calcium phosphate (CaP), hydroxyapatite (HAp), and α/β-tricalcium phosphate (α/β-tcp) are widely utilized in the preparation of multifunctional scaffold systems.Although they lack in individual antibacterial performance, they are considered as the most promising bone tissue scaffold materials due to their high biological compatibility and sufficient mechanical strength, resulting from the high similarity with the inorganic salt components in natural bone tissue.^113,114^ Therefore, it is expected to achieve bio-ceramic scaffolds with high activity antibacterial performance by selecting different antibacterial mechanisms through drug induction, ion loading, physical activation, and other methods.^115^

##### Organic polymers for bone infection

Organic antimicrobial materials refer to materials composed of organic compounds, which exhibit inherent or potential antimicrobial properties. They typically include natural organic antimicrobial materials and synthetic organic antimicrobial materials.^116^ Among natural organic antimicrobial materials, chitosan is one of the natural cationic polysaccharide antimicrobial materials, in which the cationic portion of the polymer combines with the negatively charged bacterial cell membrane, while the hydrophobic segments can insert into the bacterial cell membrane, causing leakage of cytoplasmic components.^117^ Protein antimicrobial materials represented by antimicrobial peptides are composed of amino acids connected by peptide bonds, which can interact with the bacterial cell wall or membrane, disrupting their structure and function.^118^ Polyphenolic compounds represented by tea polyphenols, curcumin, and resveratrol exhibit direct antimicrobial activity, inhibition of bacterial virulence, and synergy with antibiotics due to the abundance of phenolic hydroxyl groups in their functional groups.^119^ Antibiotics such as vancomycin, rifampicin, amoxicillin, and levofloxacin are representative synthetic organic antimicrobial materials widely used in the prevention and treatment of periprosthetic infections. However, they have drawbacks such as antibiotic dosage dependence, development of bacterial resistance due to high-dose usage, and systemic and local cellular disadvantages.^120,121^

In bone regeneration, natural polymers consisting of collagen, gelatin, alginates, chitosan, sericin, hyaluronic acid, and other components are commonly used as substrates for the preparation of 3D hydrogels.^122,123^ These polymers typically exhibit good biocompatibility, promote cell proliferation and adhesion at room temperature. The unique three-dimensional network structure provides favorable space for the loading of bioactive molecules, aiding in controlled drug release. Furthermore, the mechanical properties and degradation behavior can be regulated through chemical and physical cross-linking.^124,125^ Compared to natural polymers, synthetic polymers have advantages such as high strength, easy processing, absence of pathogenic impurities, and biodegradability. Currently, synthetic polymers successfully applied in the field of orthopedics include polyetheretherketone (PEEK), polycaprolactone (PCL), and polylactic acid (PLA). Polylactic acid is a low-cost, biodegradable renewable biomaterial. Most importantly, it is safe for the human body and can be absorbed by tissues. Combined with its excellent physical and mechanical properties, it can be used to construct bone tissue engineering scaffolds in the field of biomedical engineering. However, single-polymer scaffolds have certain limitations. Their performance is relatively singular, making it difficult to meet the multifunctional requirements of clinical applications. Various synthetic polymers, such as polycaprolactone (PCL) and polytrimethylene carbonate (PTMC), have been used to construct polylactic acid-based blend composite scaffolds for regenerating different tissues such as cartilage and bone.^126–128^

#### Preparation method of bone infection repair scaffold

Based on the performance of bone tissue substitutes, it is necessary to select a reasonable process. In the preparation of bone tissue substitute scaffolds, gas foaming, electrospinning, and particle leaching are commonly used.^129–131^ However, in an ideal situation, designing bone tissue substitute scaffolds with controllable hierarchical porous structures can provide personalized treatment plans for patients. Additive manufacturing technology (AM) has been widely developed and applied in the field of orthopedics for anatomical models, surgical instruments, and fixation devices, typically featuring high precision, speed, and automation.^99,132^ In this section, we will introduce the techniques used for the preparation of bone tissue engineering substitute scaffolds, including traditional preparation techniques and additive manufacturing techniques such as 3D and 4D printing. Partial application scenarios are listed in Table 2.^133–147^

**Table 2:** Preparation method of bone infection repair scaffold

Fabrication methods	Materials	Processing methods	pore size	mechanical strength	Culture cell	Type of bone repair	References
Fused deposition Modeling (FDM)	PEEK	BP-NS coating, photothermal effect	200 μm, 500 μm, 800 μm	745.4 MPa, 328.6 MPa and 247.9 MPa	*S. aureus*, E. coli, MG-63 cells and UMR-106 cells	Bone defect	^133^
Selective Laser Sintering (SLS)	PLLA/BG	Drug loading	450 ~ 500 μm large pore and 10 ~ 90 μm micropores	/	BMSCs	Bone defect	^134^
Digital Light Processing (DLP)	GelMA/HAp	Cross-links	5.85 mm and 50 nm	681.06 kPa and 904 kPa	MC3T3-E1 cell	Bone defect	^135^
Electrostatic spinning	Poly (3-hydroxybutyrate-co-4-hydroxybutyrate)/poly (vinyl alcohol) P34HB/PVA	Cell loading	Mean fiber diam-eters were 0.23 mm, 1.33 mm and 2.16 mm	Young’s modulus was 32.70 ± 6.88 MPa, the tensile strength was 2.93 ± 0.13 MPa and the UTS was 0.25 ± 0.15 MPa.	hBMSCs	/	^136^
Freeze drying	nHA/Collagen	Cross-links	14.3–48.7 μm(28.8 ± 7.8) and 13.9-39.7 μm(26.1 ± 5.2)	/	/	Osteoporosis bone defect	^137^
Gas foaming	Poly (propylene carbonate) (PPC)/ starch/ bioglass	/	100 ~ 500 μm	38.5 ± 1.9 MPa(50 bar), 19.24 ± 0.36 MPa(75 bar), 19.24 ± 0.36 MPa(75 bar)	Primary human dermal fibroblasts	Bone defect	^138^
Hot sintering	nHA, PHB, PCL	Single emulsion (o/w) technique prepare microspheres	34.34 nm	/	MG63 cells	Segmental bone defect	^139^
Solvent casting/Particulate leaching	PCL/HAp	/	(87.2 ± 1.7)% porosity	0.52 ± 0.12 Mpa	MG63 cells	Bone defect	^140^
Low temperature injection	PLGA/PLA/HA/CS	Nano-spray-drying technique	/	/	/	Bone infection	^141^
3D printing	VAN/PLGA/β-TCP	Electrostatic and physical crosslinking	102 μm	6 MPa	BMSCs	Infectious bone defect	^142^
3D printing	GelMA/PLA/PEG/PLA	Cross-links	500 μm	4.7 ± 1.90 kPa, 4.5 ± 1.84 kPa, 4.0 ± 1.39 kPa, 4.2 ± 2.09 kPa, 4.0 ± 1.71 kPa, 3.4 ± 1.55 kPa	BMSCs	large segmental bone defect	^143^
3D printing	PDA	Cross-links	/	≈4 MPa	MC3T3‐E1 cells	Osteoporosis bone defect	^144^
3D printing	Alg/GOx/CaP@CAT	Cross-links	≈800 μm	/	rBMSCs,HUVECs, and RAW 264.7 macrophages	Diabetic Bone Regeneration	^145^
4D printing	TCP/P(DLLA-TMC)	Photothermal effect	360 ± 40 µm	2.2 ± 0.2 to 2.4 ± 0.2 MPa (20°C), 1.5 ± 0.2 MPa(37°C), 0.4 ± 0.05 MPa(45°C)	BMSCs	Critical size bone defect	^146^
4D printing	nHA/SMPU	Cross-links	/	/	Primary fibroblasts	Cartilage defect	^147^

##### Traditional fabrication method

**(1) Electrospinning**

Electrospinning is a process in which a viscous or solution state polymer is extracted from a nozzle by electrostatic force. It typically consists of four components: an injection pump, a power source, a metal needle, and a metal collector.^148,149^ The electrospinning technique can be used to produce high-precision nanofibers and can also create pre-designed patterns based on computer-controlled movements. By directly changing the electrospinning parameters, the surface area and aspect ratio of the scaffold can be controlled, and there is often no excessive toxicity. However, the main problem is that there is repulsion between the continuously deposited fibers, and the printing thickness often cannot exceed 4 mm. The low porosity and pore size of the fibers themselves make it difficult for cells to enter and proliferate within the electrospun scaffold. Therefore, it is difficult to be the mainstream method for preparing bone tissue engineering substitutes, and it is usually used as a coating method to modify existing scaffolds.^150,151^

**(2) Gas foaming**

Gas foaming is also a technique for preparing porous scaffolds. First, the polymer is mixed into a gel and poured into a mold to solidify. Then, gas bubbles are expelled from the material through chemical or physical reactions, forming a porous structure.^152^ The main advantages of bubble foaming technology are low cost and the ability to prepare a large number of scaffolds in one operation. However, the uncontrolled bubbles result in uneven pore distribution and non-interconnected pores in the scaffold.^153^

**(3) Freeze drying**

Freeze drying is a method in which suitable solvents are used to prepare materials into liquid form, and then low-temperature freezing in molds solidifies them. Finally, the solvent is sublimated under low pressure to obtain a porous scaffold. The scaffolds produced by this preparation method can have a porosity as high as 90% and a pore size range of 20 ~ 200 μm. The porosity can be controlled by freeze drying rate, polymer concentration, and temperature.^154,155^

**(4) Solvent casting/particle leaching**

Solvent-casting particulate leaching (SCPL) is a method in which polymers are dissolved in organic solvents and then salt particles of a certain size are added to the solution and fixed in a three-dimensional mold. As the solvent evaporates, the polymer and salt particles form a composite material. Finally, the composite material is placed in a solution containing dissolved salt particles, leaving behind the polymer to form a porous scaffold.^156,157^ Nevertheless, three-dimensional scaffolds prepared using this method often suffer from insufficient mechanical strength and the presence of residual organic substances during the evaporation of solvents and dissolution of salt particles, resulting in certain cytotoxicity.^158^

##### Additive manufacturing technology

**(1) Fused deposition modeling**

Fused Deposition Modeling (FDM) technology is a process that involves heating thermoplastic material and, under the control of a computer-aided system, extruding continuous filaments of material onto a worktable according to a pre-set model. The first layer of the model solidifies after rapid cooling. Subsequently, the nozzle rises and repeats the extrusion process on the existing layer, stacking a new layer to build a three-dimensional structure. The main advantages of this technology are its excellent mechanical performance, high precision printing capability, and low-cost manufacturing. However, its main limitation is that it can only be used with thermoplastic polymer materials as the printing substrate.^159,160^

**(2) Selective laser sintering**

Selective Laser Sintering (SLS) is a process in which material powder is spread on the surface of a formed part. By controlling the laser beam to scan the powder according to the cross-sectional contour, bonding is formed between the powders when the temperature rises to the melting point. This process is repeated layer by layer on the working platform to ultimately create a complete three-dimensional solid structure.^161^ It usually uses materials such as metal and polymer as printing powders, and it can achieve a printing accuracy of 20 ~ 50 μm.^162^

**(3) Stereo lithography appearance**

Stereo lithography appearance (SLA) is one of the earliest 3D printing technologies applied in the field of skeletal engineering. During the printing process, ultraviolet light is projected onto photosensitive resin using the principle of light crosslinking to construct three-dimensional scaffolds with high resolution and precise shape. Clear interconnected pores and uniform pore sizes can be observed inside the scaffold.^163^ Although SLA has been used for the preparation of bone repair scaffolds, the light-curing materials used have poor degradation rate and biocompatibility.^164^

**(4) 3D Bioprinting**

3D bioprinting is based on additive manufacturing, which utilizes live cells, composite materials, and additional components such as growth factors to prepare bio-inks. These bio-inks are then deposited on a workstation through computer-aided construction, forming a three-dimensional structure and creating a scaffold for bone tissue engineering.^165,166^ Biological ink can be classified into cell-loaded biological ink and non-cell-loaded biological ink. Among them, cell-loaded biological ink typically exhibits not only good printing performance but also provides a favorable growth environment for cells.^167^ Biological ink can be composed of natural polymer compounds such as gelatin, sodium alginate, and fibrous protein, which simulate the extracellular matrix (ECM).^168,169^ It can also be printed using bio-ceramic materials as substrates. Synthetic polymers such as PCA, PLA, and PTMC are also suitable for printing with biological ink.^170,171^ During the printing process, the selection of appropriate printing parameters is equally important due to the fact that biological inks are composed of multiple materials and carriers. For instance, the nozzle is often clogged by cells or materials with larger particle sizes, which affects the extrusion of the biological ink. Additionally, during the extrusion process, considerations need to be given to shear stress, thermal stress, and cell integrity. Temperature-sensitive materials such as gelatin and fibroin can be influenced by temperature, leading to changes in the rheological properties of the ink. Furthermore, under high extrusion pressure, cell viability is also reduced.^172–174^ The three-dimensional structure of bone tissue and the extracellular matrix of bone cells is a dynamic process, accompanied by constant decomposition and synthesis. Therefore, traditional 3D biotechnology that only stays in the initial state of printing cannot simulate this dynamic change process.^175,176^ Therefore, 4D printing technology enables bioactive scaffolds to respond to various dynamic changes over time, such as changes in pH, temperature, light, electricity, and other chemical and physical changes.^177^ Among them, shape memory materials (SMM) are a special type of stimulus-responsive material in 4D printing. They can affect the memory of their macroscopic shape under various physicochemical stimuli. Generally speaking, the size and shape of bone defects vary. Therefore, shape memory scaffolds in 4D printing can help adjust the scaffold to the appropriate shape to fill the void in the bone defect.^178,179^ Hu et al. used PLLA-TMC to prepare a shape memory scaffold. The scaffold can be temporarily fixed at low temperatures and completely recover its original shape at simulated body temperature (37 °C). They also evaluated the biocompatibility of the scaffold with BMSCs and found that the cells exhibited good attachment under the scaffold.^180^

#### Composite scaffold system for bone infection repair

An ideal scaffold system for bone infection repair should couple infection treatment with defect regeneration. In previous studies, we mentioned the selection of materials with intrinsic bone regeneration or intrinsic antibacterial capabilities. Additionally, different modes of scaffold preparation systems can be chosen based on the specificity and complexity of the patient’s injury site, thus achieving personalized treatment.^96^ Therefore, a rational selection of combining different characteristics of substrates as a means to promote the mechanism of infection treatment and bone regeneration, along with the selection of a rational scaffold preparation method, can achieve an “all-in-one” treatment approach, avoiding multiple surgeries and overcoming the traditional challenges of repairing bone infections and defects.^181^

##### Hydrogel scaffold

Hydrogels are soft-tissue-like materials with high water content, and their injectable characteristics allow for direct injection into bone defect sites to fill cavities and provide a scaffold for the growth of new bone tissue.^182^ At the same time,the hydrogel encapsulates drugs within it, forming a stable drug-carrying system which enable the release of antimicrobial agents within the infected foci, reduce the risk of infection, and promote bone regeneration. The preparation methods of injectable hydrogel systems mainly include physical cross-linking and chemical cross-linking. Physical cross-linking is the formation of hydrogels by linking polymer molecules together through physical interactions, such as electrostatic interactions, hydrogen bonding, and van der Waals forces. Hydrogels prepared by this method usually have better stability and biocompatibility, but their mechanical strength and injectability may be poor. Chemical cross-linking is used to form hydrogels by introducing chemical cross-linking agents, such as aldehydes, amides, and esters, to form covalent bonds between polymer molecules. Hydrogels prepared by this method usually have better mechanical strength and injectability, but their preparation process is more complicated and may have some effect on the biocompatibility of hydrogels.^183^ In addition, there are some novel preparation methods, such as photocrosslinking and click chemistry, that can prepare hydrogels with specific properties, such as tunable mechanical strength and degradability.^184,185^

By regulating the nature and structure of hydrogels, the slow release rate and dosage of drugs or growth factors can be controlled to achieve individualized therapeutic regimens.^186^ Additionally, hydrogels can protect drugs or growth factors from rapid decomposition or excretion after injection, improving bioavailability and efficacy.^187^ The slow-release properties of hydrogels can currently be modulated by selecting appropriate materials, such as polymers and natural biomaterials, based on the characteristics of the target substance being released.^188^ Furthermore, the design of hydrogels with responsive properties, such as temperature, pH, or stimuli-responsive hydrogels including electromagnetic fields, can enable modulation of release rates based on the external environment.^189^

In the treatment of bone infections, hydrogels play a crucial role in releasing bioactive molecules into the surrounding tissues. Through diffusion and osmosis, these molecules exert their biological effects.^190^ Hydrogels can be loaded with biologically active molecules such as antibiotics and growth factors, effectively inhibiting the growth and reproduction of bacteria while promoting the repair and regeneration of bone tissues, thereby alleviating bone infections.^191^ This chapter reviews the research progress in bone scaffolds loaded with active molecules for anti-inflammatory and antibacterial purposes, with a specific focus on the use of injectable hydrogels. The active biomolecules reviewed include stem cells and growth factor nanoparticles, and the chapter analyzes the loading methods, release mechanisms, and biological effects associated with these active molecules within the hydrogel

**(1) Stem cells loaded with hydrogel scaffolds**

The properties of hydrogels promote cellular responses and cellular distribution at any site prior to the transition phase leading to gelation. Due to their similar structural properties, the heavily hydrated nature of hydrogels allows for significant mimicry of the extracellular matrix (ECM). This provides an ideal environment for cellular regeneration and proliferation, enabling cells encapsulated in the hydrogel to grow and secrete new ECM for restoring damaged tissue.^192^ When exogenous cells are implanted in a bone defect area, they often undergo immune rejection and face adverse microenvironments such as inflammation and hypoxia. These factors can lead to a large number of implanted cells dying within a short period of time, ultimately affecting the speed and efficacy of bone repair. The repair and functional reconstruction of bone injuries heavily rely on cells, and stem cells have been shown to play a crucial role in this process.^193^ Endogenous stem cells, which are pluripotent cells located in specific tissues or circulating in the bloodstream, possess the abilities of self-renewal and differentiation into various cell types.^194^ The use of endogenous stem cells offers advantages over exogenous stem cells, including reduced risks of immune rejection and infection. Osteogenic bone marrow mesenchymal stem cells (hMSCs) are commonly used in bone regeneration to promote the differentiation of osteoblasts and the mineralization of the extracellular matrix. However, the clinical application of direct hMSC injection has been limited by low cell retention and implantation rates, thereby necessitating the development of suitable cell delivery scaffolds. These scaffolds can protect cells from environmental stresses and maintain their retention in damaged tissues.^195^ Bastami et al. employed a 3D-printed biodegradable hydrogel composed of alginate, gelatin, and freeze-dried bone allograft nanoparticles (npFDBA) as a scaffold to enhance the adhesion, proliferation, and osteogenic differentiation of rat bone marrow mesenchymal stem cells (rBMSCs). The behavior of rBMSCs on the scaffolds was evaluated using scanning electron microscopy, MTT assay, and qPCR. The results demonstrated excellent adhesion, proliferation, and differentiation of rBMSCs on the 3D printed hydrogels. Furthermore, hydrogels loaded with rBMSCs exhibited comparable new bone regeneration to the FDBA group loaded with rBMSCs (*P* < 0.05). These findings were confirmed by Masson’s trichrome staining and osteocalcin expression. Therefore, 3D printed hydrogels loaded with rBMSCs have the potential to significantly enhance bone regeneration, surpassing traditional clinical approaches (FDBA).^196^ Compared to bone marrow MSCs, a large number of adipose-derived stem cells (ASCs) can be easily isolated through liposuction. ASCs secrete various factors and cytokines that promote osteoinduction and angiogenesis. However, ASCs have limited capacity for osteogenesis and bone healing. The combination of ASCs with growth factors like BMP2 has shown improved healing and reduced bone infections.^196^ In a study conducted by Tang et al., an injectable and in situ crosslinked gelatin microribbon (μRB)-based macroporous hydrogel was prepared using the wet spinning method. The injectability was optimized by adjusting the glutaraldehyde concentration for internal cross-linking, and μRB shapes were encapsulated with fibrinogen. The efficacy of this injectable μRB scaffold in supporting ASC delivery and promoting bone regeneration was evaluated using a mouse cranial defect model. ASC survival was assessed through bioluminescence imaging, bone regeneration was evaluated through microCT, and degradation and biocompatibility were determined through histologic analysis. The researchers first optimized the injectability of the gelatin μRB by varying the glutaraldehyde concentration. They then coated the injectable μRB preparation with fibrinogen, allowing for in situ cross-linking via prothrombin. Fluorescence imaging and histologic examination revealed that most μRBs were degraded after 3 weeks. Immunostaining showed that M1 macrophages were recruited to the defect at day 3, and were later replaced by M2 macrophages by week 2. This study demonstrated that μRB-based scaffolds enhanced ASC survival and accelerated bone regeneration when injected into mice with critical-sized cranial defects. The injectable μRB scaffold has the potential to be a versatile biomaterial for delivering various types of stem cells and promoting tissue regeneration.^197^ Embryonic stem cells (ESCs) have the ability to promote osteogenic differentiation, mineral deposition, and production of mesenchymal stem cells.^198^ In addition, microspheres are often limited by structural heterogeneity, non-uniform size, low cell loading capacity, and poor cell viability.

**(2) growth factors loaded on hydrogel scaffolds**

Growth factors are biomolecules that can stimulate cell proliferation and differentiation to promote tissue growth and repair. Injecting growth factors directly into the region of damaged bone tissue can activate the growth and differentiation of surrounding cells, thus accelerating the process of bone tissue regeneration and repair.^199^ Commonly used growth factors include bone morphogenetic protein (BMP), platelet-derived growth factor (PDGF), and fibroblast growth factor (FGF).^195,200^ Often, growth factors are used in combination with other bioactive molecules to better promote osteogenesis, thereby accelerating the recovery and repair of bone tissue.^201^ Ratanavaraporn et al. evaluated the effectiveness of gelatin hydrogels doped with a combination of stromal cell-derived factor-1 (SDF-1) and BMP-2 on the regeneration of bone from rat ulna adventitial defects and subcutaneous sites. The researchers observed similar release profiles of SDF-1 and BMP-2 from the hydrogels, and the hydrogels containing both SDF-1 and BMP-2 enhanced bone regeneration. Furthermore, when hydrogels incorporating SDF-1 and BMP-2 were implanted, increased expression levels of the Cxcr4, Runx2, and osteocalcin genes were observed. Experiments in green fluorescent protein-positive chimeric mice showed the formation of vascular-like structures and a significant accumulation of cells positive for CD1 and CD2 at the site of implanted hydrogels with a combination of SDF-1 and BMP-2. Additionally, a large fraction of nonhematopoietic cells positive for CD29 and CD44 were detected. The combined release of SDF-1 and BMP-2 enhanced the recruitment of osteoblasts and angiogenesis, resulting in a synergistic effect on bone regeneration.^202^

**(3) Nanoparticles loaded on hydrogel scaffolds**

Nanoparticles promote the proliferation and differentiation of osteoblasts and enhance bone regeneration and can be incorporated into hydrogels as bioactive substances, such as calcium ions and phosphates.^203^ Among the nanomaterials used in bone tissue engineering, superparamagnetic Fe_3_O_4_ nanoparticles (MNPs) and hydroxyapatite (HAP) nanoparticles are commonly employed. These materials have been shown to improve bone tissue mineralization when incorporated into injectable hydrogel systems.^204^ Posadowska et al. directly doped gentamicin into a knotted coolant hydrogel and embedded the hydrogel in nanoparticles. The drug exhibited an expected burst release within the first 12 h of injection, with doses reaching approximately 27% of the total gentamicin. This was followed by a gradual and sustainable release, with gentamicin levels reaching approximately 90% of the initial dose within 60 days. In vitro studies confirmed the antimicrobial activity of the system against Staphylococcus spp., as well as its cytocompatibility with osteoblast-like cells.^205^ Wassif et al. prepared composite in situ-forming hydrogels of chitosan at three different concentrations. They also prepared spray-dried PLGA/PLA nanoparticles loaded with linezolid using the nanospray-drying technique. Water-soluble carriers (PVP K30) and fat-soluble carriers (cetostearyl alcohol) were used, along with three copolymers (DL-propionate and/or DL-propionate-co-glycolide), to optimize the preparation of the linezolid nanoparticles. The optimized linezolid nanoparticles were then incorporated into the optimized composite hydrogels containing nanohydroxyapatite (nHA). The combined hydrogel/nanoparticle system exhibited good injectability at 37°C, and the optimal ratio resulted in a sustained release of linezolid for 7–10 days. This suggests that linezolid can reduce the frequency of injections and improve patient compliance during the treatment of bone infections. The injectable system of chitosan in situ molded composite gels combined with biodegradable nanoparticles loaded with linezolid has shown promising results in the long-term treatment of bone infections.^141^ Tao et al. designed a thermosensitive hydrogel based on chitosan (CS) for the production of VCM nanoparticles (NPs)/gel local drug delivery systems. They used quaternary chitosan and carboxylated chitosan nanoparticles (VCM-NPs) as raw materials to form VCM-NPs through positive and negative charge adsorption. This approach was aimed at improving the encapsulation efficiency and drug loading of VCM, as well as preventing infections and promoting fracture repair. The hydrogel was assessed in a rabbit osteomyelitis model.^191^

##### Solid bone tissue substitute

Injection-type hydrogels are favored for small-range defects due to their simple operation, minimal trauma, and good filling effect. However, as a chronic disease requiring long-term treatment, osteomyelitis often causes defect spaces to expand during debridement and requires the filling material to bear stress at both ends of the defect. Solid scaffolds typically have characteristics such as shape stability, complex structure, and diverse functions. Most importantly, they can provide mechanical support that matches the mechanical properties of bone tissue through material optimization. Therefore, in strategies for treating infectious bone defects, functional integrated solid scaffolds are usually selected for filling and treatment. Among them, 3D printing, as mentioned earlier, can create biomimetic bone repair scaffolds based on precise design of filling rate, scaffold structure, size, and shape.^206^ In this chapter, we focus on reviewing the application of 3D printed solid scaffolds in bone infection, as well as the incorporation of active factors, stem cells, and drugs. Other preparation techniques such as electrospinning and freeze-drying will be discussed in Table 2 and Fig. 3.

**Fig. 4:**
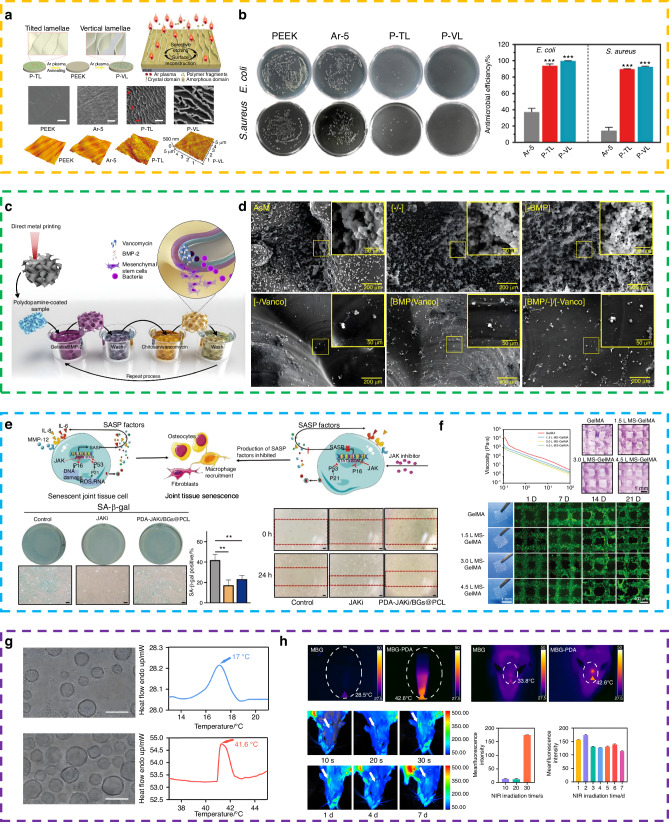
Solid bone tissue substitute. **a** Physical modification method was used to construct nano layer on the surface of the PEEK. **b** Antimicrobial efficiency and pictures of the bacterial colonies after different treatments. Reprinted with permission from ref. 212, Copyright 2023 Materials Horizons **c** The surface of the material was modified by BMP and vancomycin layer-by-layer coating. **d** CFUs of planktonic and adherent bacteria measured for the different experimental groups. Reprinted with permission from ref. 214, Copyright 2020 Additive Manufacturing **e** 3D scaffold loaded growth factors to enhance the activity of 18 M BMSCs by inhibiting SASP through JAKi treatment. Reprinted with permission from ref. 221, Copyright 2023 Advanced Healthcare Materials **f** 3D bioprinting bioceramic scaffolds containing HUVECs. Reprinted with permission from ref. 241, Copyright 2023 Advanced Healthcare Materials **g** Scaffolds were loaded with liposomes. **h** The photothermal effect of scaffolds and the controllable release of payloads from liposomes or platforms in vitro and in vivo. Reprinted with permission from ref. 227, Copyright 2023 Bioactive Materials

However, most solid implants require modification of the surface itself to address bacterial infection and inflammatory response in bone infection, as well as bone regeneration. This modification mainly includes physical and chemical methods to modify the surface, avoiding bacterial adhesion, killing bacteria, and reducing the formation of biofilms.^207–209^

The physical modification of the surface of the implant significantly affects hydrophobicity, van der Waals forces, and electrostatic interactions, thereby influencing bacterial adhesion to the implant surface. Additionally, physical modification can lead to the appearance of nanostructures such as nanofibers, nanotubes, or nanoneedles, which can penetrate bacterial cell membranes and cause cell death.^210,211^ The size, dimensions, aspect ratio, and mechanical properties of these nanostructures play a crucial role in their antibacterial efficacy. Furthermore, light-activated surfaces, represented by photothermal therapy (PTT) and photodynamic therapy (PDT), can respond to external light stimulation and exhibit antibacterial properties. Wang et al. used plasma treatment, such as argon plasma, to prepare tilted and vertical nanostructured layers on semicrystalline polyetheretherketone (PEEK) and systematically investigated the in vitro and in vivo antibacterial and osteogenic properties of these vertical and tilted nanostructures, as well as their antibacterial mechanisms (Fig. 4a, b).^212^

Unlike physical modification methods on the surface of materials, chemical modification on the surface of implants is usually achieved through coating and chemical bonding. This process involves the incorporation of bioactive molecules with antibacterial and anti-inflammatory properties, such as antibiotics, peptides, and metal ions. By disrupting bacterial adhesion to biomaterials, this approach provides a unique therapeutic method for bone infections.^213^ Yavari et al. proposed a layer-by-layer modification approach, where they first prepared a three-dimensional titanium-based metallic scaffold with a biomimetic topological structure using a selective laser melting (SLM) technique. They then coated the scaffold surface with bone morphogenetic protein-2 (BMP-2) and vancomycin using chitosan and gelatin-based shells with cationic and anionic groups, respectively. In vitro drug release testing showed that this multilayer coating approach delayed the release of vancomycin and BMP-2 for 2-3 weeks. Under the action of vancomycin, the [BMP/-]/[-/Vanco] group completely eliminated the adherent bacteria on the first and seventh days (Fig. 4c, d).^214^

**(1) Solid bone substitutes are loaded with growth factors**

Growth factors play an important role in the repair of bone defects, regulating cell proliferation, differentiation and migration. Common growth factors include bone morphogenetic protein (BMP), vascular endothelial growth factor (VEGF), fibroblast growth factor (FGF), and transforming growth factor-β1 (TGF-β1).^215^ BMP growth factor can stimulate the differentiation of bone progenitor cells, chondrocytes and osteoblasts and promote the formation and functional recovery of new bone tissue. Bmp-2 and BMP-7 are approved by the FDA for clinical treatment.^216,217^ To ensure the sustained release of BMP-2 in the body, Park et al. prepared a unique leaf-stacked structure (LSS) microsphere and encapsulated BMP-2 in it. Finally, the entire leaf-stacked structure (ELSS) was sealed with SA bioink, and a three-dimensional structural scaffold was formed using medical-grade polycaprolactone (mPCL) through low-temperature 3D printing. Two concentrations of BMP-2 (1 μg/mL and 10 μg/mL) were used to test the sustained release effect of the ELSS structure on growth factors. The results showed that after 26 days, BMP-2 was released slowly, with the release amount of 1 μg/mL BMP-2 accounting for 79% of the total encapsulation amount [(66.4 ± 0.5 ng)/10 mg] and the release amount of 10 μg/mL BMP-2 accounting for 51% of the total encapsulation amount [(313.7 ± 0.2 ng)/10 mg] particles. Runx2 is a known specific marker for osteogenic differentiation. The intensity of the signal was observed by staining with a Runx2 antibody, and the green fluorescence (RUNX2) was more pronounced in the ELSS1 group than in the ELSS0 group. This indicates that the continuous supply of BMP-2 promotes osteogenic differentiation of hPDCs and biomineralization. The immune microenvironment of bone injury is crucial for bone tissue regeneration. In different signaling microenvironments, macrophages can polarize into the proinflammatory M1 phenotype, stimulating inflammation and fibrosis, or polarize into the pro-healing M2 phenotype, aiding in stem cell osteogenesis and ECM remodeling for bone repair.^218,219^ As mentioned earlier, M2 macrophages can enhance the bone regenerative microenvironment by stimulating the bone growth factors BMP-2 and VEGF. Therefore, Liu et al. promoted healing in bone injuries by creating an immune microenvironment through the use of the immune-regulating cytokine interleukin-4 (IL-4). Specifically, they prepared a 2D heterogeneous nanostructure composed of graphene oxide (GO) layers and black phosphorus (BP) nanosheets, which were then coated on the surface of a 3D-printed PCL polymer scaffold to create a 3D-printed multifunctional scaffold with a 2D heterogeneous nanostructure (3D-Scaf-GOBP-Immuno). To impart immunization functionality to the scaffold, the 3D-Scaf-GOBP-Immuno was immersed in a rat IL-4 solution and achieved recruitment and adsorption of IL-4 through its unique 2D structure. In the immunofluorescence images, compared to other scaffolds, the 3D-Scaf-GO-Immuno and 3D-Scaf-GOBP-Immuno scaffolds exhibited more macrophage aggregation on their surfaces. Additionally, changes in macrophage phenotype on the 3D scaffold were validated, and immunolabeling was performed using CD68 and CD206 (a specific marker for M2 macrophages). The results showed that CD206 macrophages were detected in the IL-4-loaded scaffolds, indicating that IL-4 loading induced macrophage polarization toward the M2 phenotype. This further confirms that the 2D heterogeneous nanostructure successfully recruited IL-4 cytokines and supported their adhesion. To explore whether the immune-functionalized 3D scaffold affects the osteogenic performance of the macrophage response, the released culture medium of macrophages was cocultured with rBMSCs. Real-time fluorescence quantitative PCR showed that the immune-functionalized scaffold significantly enhanced the mRNA expression of osteogenic markers, including OPN, OCN, Runt-related transcription factor 2 (Runx2), and OSX. Confocal images further demonstrated intense expression of OPN protein on the 3D-Scaf-GOBP-Immuno scaffold by rBMSCs. Combined with subsequent in vivo experimental analysis, this immune-functionalized scaffold can release and deliver IL-4 cytokines from the surface of the 3D scaffold to adjacent tissues, stimulating M2 macrophage polarization and bone immune regulation. Additionally, graphene oxide (GO) improves cell adhesion, and the synergistic effect of sustained release of phosphate and IL-4 effectively induces neoangiogenesis and osteogenesis at the bone site, accelerating bone repair.^220^ Li et al. simulated the layered biomimetic nanostructure of extracellular matrix (ECM) to prepare a three-dimensional electrospun scaffold (pd-jaki/BGs@PCL). Mesoporous bioactive glass nanoparticles (BGs) were used to encapsulate a JAKi (ruxolitinib), and then the JAKi-encapsulated BGs (JAKi/BGs) were uniformly dispersed in poly(ε-caprolactone) (PCL) nanofibers. In subsequent in vitro studies, it was found that the composite scaffold effectively suppressed the expression of aging biomarkers (P16, P21, and P53) and senescence-associated secretory phenotype (SASP) markers (IL-6, IL-8, MMP12, and PAI-1) (Fig. 4e) and showed promising potential in treating age-related bone diseases.^221^ Furthermore, the combined use of growth factors and other bioactive molecules often produces better results, especially in addressing the eradication of bacteria, inhibition of inflammation, and bone resorption that are needed in the treatment of bone infections.^222^

**(2) Solid bone substitutes are loaded with nanoparticles**

Mesoporous bioactive glass (MBG) is a biocompatible nanoparticle primarily composed of silicon dioxide. It is often used for the adsorption or encapsulation of a large amount of bioactive molecules due to its high surface area and pore volume. Based on MBG, Sánchez et al. designed mesoporous glasses (MGNs) with a radial mesoporous structure and a size range of 20 to 150 nm. They encapsulated both antibacterial and anti-inflammatory zinc ions and curcumin in MGNs (MGN-ZnCur) and achieved controlled release targeting using the specific functionality of active -OH groups on the surface of MGNs. The release of three types of ions, Ca^2+^, P, and Zn^2+^, in α-MEM medium showed that the release of Zn ions was the highest, reaching 22 × 10^-6^ at 24 h and (23–24) × 10^-6^ at 72 h. In subsequent antibacterial experiments, the antibacterial ability of the MGNs + zinc + curcumin composite system was improved, and the growth rate of bacteria significantly decreased at concentrations of 125 and 150 μg/mL. The qualitative results of the biofilm degradation test and the coculture experiment of *S*. *aureus* and preosteoblast MC3T3-E1 cells using MGNs loaded with curcumin at a concentration of 150 μg/mL also revealed that the encapsulation of zinc and curcumin could prevent biofilm formation and promote the proliferation of MC3T3-E1 osteoblasts.^223^ MBG nanoparticles are also suitable for encapsulating various types of active biomolecules. Sui et al. prepared a nanocomposite material (MSN-BMP4-EN) that encapsulates the highly efficient bone-inducing growth factor BMP-4 and broad-spectrum antibiotic norfloxacin (EN) to inhibit bacterial and osteoclast proliferation and improve bone regeneration during infection. In vitro evaluation showed that MSN-BMP4-EN promoted bone differentiation by enhancing the expression of protein genes such as ALP, BMP, BSP, OCN, OPN, and RUNX-2 mRNA. It also verified the differentiation of BMSCs through the SMAD signaling pathway. Moreover, MSN-BMP4-EN inhibited the expression of TRAP, NFATc6, CTSK, and DC-STAMP, thereby impeding the excessive generation of early osteoclasts. Furthermore, MSN-BMP4-EN effectively suppressed the secretion of proinflammatory mediators, counteracting the inflammation induced by infection and promoting bone regeneration.

Liposomes, due to their structural similarity to cell membranes, play a significant role in drug delivery for bone regeneration. They have the following advantages: (1) physical encapsulation enhances drug stability and increases dosage; (2) the amphiphilic molecular layer of liposomes can be modified through physical and chemical means to achieve targeted tissue binding; and (3) altering the surface charge of liposomes allows for the delivery of DNA and RNA drugs.^224–226^ In the study of bone regeneration, Che et al. encapsulated the osteoporosis-inhibiting peptide teriparatide-like peptides (TSLs) in liposomes and successfully adsorbed them onto mesoporous bioactive glass scaffolds with biomimetic bone microstructure (TSL/PDA/MBG) through the “mussel” effect of polydopamine (PDA) (Fig. 4g, h). This achieved precise local release of the TSL peptides through the inspiration of a photothermal response and the strong adhesive coating of PDA. Regarding the thermal responsiveness of TSL/PDA/MBG, the release behavior of liposomes was tested using calcein green. The results showed that a large amount of labeled TSL liposomes were released in the solution at 42 °C, while only a small amount of calcein green precipitated at room temperature (37 °C), indicating the successful achievement of controlled release of TSL liposomes with a high transition temperature. The osteoporosis model was established in female rats by bilateral ovariectomy, and the repair of cranial defects was observed. The defect site was reconstructed in 3D using micro-CT, and after 4 weeks, the sham operation group still had a large cavity at the defect site, indicating that osteoporotic rats cannot repair cranial defects on their own. Meanwhile, without the release of TSL liposomes or the simple injection of teriparatide, the healing effect of the bone defect was still slow. The TSL/PDA/MBG scaffold group showed the best promotion of bone defect healing behavior. Finally, for systemic osteoporosis, the TSL liposome-loaded scaffold exhibited good bone regeneration ability in the femur and lumbar vertebrae.^227^ Toita et al. developed a method using artificial apoptotic cell mimetics (phosphatidylserine liposomes; PSL) to polarize M1 macrophages toward an M2 phenotype while promoting the conversion of bone homeostasis and facilitating bone tissue regeneration. In the macrophage response test on a titanium substrate, macrophages induced by LPS on the Ti surface exhibited a typical M1 inflammatory cell phenotype and secreted the proinflammatory cytokines IL-6α and TNFα. However, with an increase in the amount of PSL, the levels of inflammatory cell factors in the PSL-coated samples were significantly reduced. This indicates that PSL-coated titanium promotes the polarization of M1 to M2-like macrophages, achieving a transition from a proinflammatory to an anti-inflammatory state.

**(3) Solid bone substitutes are loaded with exosomes**

Exosomes are a type of extracellular matrix component that is secreted by cells and widely present in biological fluids and cell media. These small structures (30–150 nm) can carry nucleic acids, proteins, and cellular metabolites, thereby playing a role in intercellular signaling pathways.^228,229^ Studies have shown that extracellular vesicles present in the microenvironment of bone defects can be engulfed by target cells such as osteoprogenitor cells and osteoblasts, thereby participating in the regulation of bone regeneration. Additionally, as a component of the extracellular matrix, extracellular vesicles can promote the osteogenic differentiation of MSCs and enhance the expression of related osteogenic proteins such as RUNX2, COL1A1, OPN, and ALP. MicroRNAs (miRNAs) are commonly found active substances in extracellular vesicles that can regulate the expression of genes related to bone formation.^230,231^ Research has indicated that extracellular vesicles contain upregulated osteogenic miRNAs (Hsa-miR-146a-5p, Hsa-miR503-5p, etc.) while activating the PI3K/Akt and MAPK signaling pathways.^232^ EVs can also play a regulatory role in inflammation. For example, extracellular vesicles derived from MSCs can promote macrophage polarization toward the M2 phenotype, leading to the expression of the anti-inflammatory factors IL-6 and TNF-α. Additionally, MSC-derived extracellular vesicles can promote M2 polarization of macrophages through the NF-κB pathway.^233,234^ Therefore, loading exosomes can regulate the bone defect microenvironment through osteogenesis and inflammation modulation. Wang et al. utilized exosomes derived from MSCs, which have immunomodulatory potential, along with S-nitrosoglutathione (GSNO) to coregulate bone regeneration. First, a polycaprolactone (PCL) scaffold with good biocompatibility was 3D printed. Then, the prepared GSNO and MSC-derived exosomes were loaded onto the PCL scaffold. The authors first characterized the isolation of exosomes, observing typical cup-shaped exosome morphology using transmission electron microscopy (TEM). The fluorescent marker PKH67 was used to label exosomes and incubated with either RAW 264.7 cells or hBMSCs. In RAW 264.7 cells, the fluorescence intensity gradually increased, and internalized exosome structures were observed in hBMSCs, demonstrating the successful isolation of exosomes from MSCs. RT‒PCR was used to test the expression of inflammatory genes, and the results showed that the addition of GSNO and exosomes reduced the gene expression of IL-6, TNF-α, iNOS, and IL-1β by five-fold, 2.5-fold, 1.6-fold, and 2.3-fold, respectively. Moreover, typical M2 macrophage morphology was observed in the subsequent scanning electron microscopy (SEM) analysis. The synergistic effect of GSNO and exosomes also showed a significant pro-osteogenic effect in the osteogenic differentiation test.^235^ Wu et al. combined exosomes secreted by Schwann cells (SCs) with porous Ti6Al4V scaffolds to observe their effects on bone regeneration and repair in vivo. In immunofluorescence staining, BMSCs in the Exos H group and Exos L group showed more filamentous pseudopodia, accompanied by tighter cell‒cell connections. Additionally, a large amount of extracellular matrix (ECM) was observed on the cell surface in the Exos H group, which is crucial for maintaining homeostasis. In the wound healing experiment, BMSCs in the Exos H group exhibited the best invasive ability, demonstrating that exosome therapy derived from SCs can enhance the motility and migration of BMSCs and effectively improve bone formation and regeneration at the site of bone defects.^236^

**(4) Solid bone substitutes are loaded with stem cells**

Mesenchymal stem cells (MSCs) are a type of multipotent adult stem cell with self-renewal ability. They can differentiate into various cell types, such as chondrocytes, adipocytes, and osteocytes. MSCs have low immunogenicity, fast in vitro proliferation, and no toxic side effects. Currently, they have been widely used in clinical practice. The most common method for tissue engineering and culturing stem cells is through 3D scaffolds. Under suitable conditions, cells proliferate, differentiate, and secrete extracellular matrix (ECM) molecules, guided by the three-dimensional space of the scaffold. This process promotes different signal transduction pathways, providing an ideal therapeutic approach for tissue repair.^237–239^ Based on previous research on 3D-printed calcium phosphate scaffolds, Wang et al. loaded peripheral blood-derived mesenchymal stem cells (PBMSCs) and endothelial progenitor cells (PBEPCs) into a 3D-printed biphasic calcium phosphate (BCP) scaffold using tissue engineering techniques. They also coated the scaffold surface with a layer of highly biologically active nanohydroxyapatite (nHA) to prepare a composite scaffold (nHA/BCP). The addition of PBMSCs, which are derived from peripheral blood, promoted the formation of vascular networks in critical-sized bone defects. By optimizing the coculture ratio of PBMSCs and PBEC to 75:25, the expression of osteogenic and angiogenic markers was maximized. Microfil angiography was performed on experimental rabbits at 6 and 12 weeks, and the late-stage vascular imaging system revealed that the BVV/TV in the nHA/BCP-PBEPC/PBMSC group was significantly higher than that in the other treatment groups (*P* < 0.01). Abundant neovascularization surrounding the scaffold could be observed. In addition, histomorphological analysis of new bone formation and mineralization was conducted using fluorescent markers such as tetracycline and calcein. The results showed that the new bone ingrowth distance in the nHA/BCP-PBEPC/PBMSC group was significantly higher than that in the other three groups (*P* < 0.05).^240^ Due to the high vascularity and innervation of most organs and tissues in the human body, the use of scaffolds loaded with vascular endothelial cells has shown great potential. Wu et al. successfully developed a cell scaffold promoting angiogenesis by combining Li-Mg-Si (LMS) bioceramic material with vascular endothelial cells through three-dimensional bioprinting technology (Fig. 4f).^241^ Liu et al. designed a multifunctional cell-laden scaffold based on a dual-phase structure of inorganic/organic materials using a multichannel printing technique. The scaffold consisted of hydroxyapatite (HA)-based self-setting calcium phosphate cement (CPC) as the rigid inorganic phase and a functionalized alginate-methylcellulose complex as the organic phase. This interactive spatial structure allowed the preparation of a bioink containing both mesenchymal stem cells (MSCs) and human osteoblasts (hOBs). The study focused on investigating the proliferation, differentiation, and migration levels of cells in the organic and inorganic phases. In both the AlgMC and AlgMC + EW results, MSCs exhibited high vitality and uniform distribution due to the structural differences between the two channels. Furthermore, the overall intertwined structure formed by CPC and the bioink chains created a wide range of pores, which is expected to ensure nutrient supply, waste excretion, and potential vascularization. In the high-magnification images of the live/dead fluorescence channel, MSCs were observed to grow along the CPC and AlgMC + EW fibers and cover the intersection between the bioink and CPC. On the 21st day, extensive migration of MSCs toward the CPC layer was observed, and by day 49, MSCs were fully enriched on the surface. Therefore, the incorporation of stem cells provides an effective method for bone regeneration integration.^242^

## Foresight

In the treatment of osteomyelitis, the eradication of pathogenic microorganisms is the foremost consideration. Although we have made many advancements in antimicrobial strategies for antibiotics in the past half-century, there has been no qualitative leap in the development and application of antibiotics, and the existing clinical care methods have not fundamentally changed. In fact, antimicrobial strategies should not only focus on the bactericidal effect of antibiotics but should also comprehensively understand the pathogenic mechanisms of microorganisms.

Specifically, in future research on bone infections, we need to focus on the following aspects: (1) To deeply reveal the interaction between the pathogen and the host, including the pathogen’s adhesion, invasion, survival mechanisms in bone tissue, and immune evasion mechanisms; (2) To explore the immune response during infection, including the recruitment and activation of inflammatory cells, and the molecular mechanisms of immune cells in bone resorption and bone formation processes; (3) To thoroughly analyze the common resistance mechanisms in bone infections, including the role of bacterial pumps, genetic mutations, horizontal gene transfer, and host genetic susceptibility. In the field of tissue engineering, we should focus on: (1) Using materials science and engineering technology to construct biomimetic 3D printed degradable scaffolds with structure, composition, and mechanical properties; (2) Achieving controlled release of antimicrobial drugs through nanocarriers or scaffold surface coating technologies; (3) Using coaxial printing or gradient printing techniques to achieve graded controlled release of antimicrobial drugs and osteogenic active substances.

In summary, by integrating frontier research at the molecular level with advanced technology in tissue engineering, innovative strategies and techniques for the treatment of bone infections have been proposed. This multidisciplinary cross-approach not only opens new therapeutic avenues for the treatment of bone infections but also promotes the development of treatment methods in this field towards more efficient and precise directions. Through this comprehensive research perspective, we hope to achieve significant breakthroughs in the treatment of bone infections in the future, thereby improving the treatment outcomes and quality of life of patients.
